# The Role of Dietary Intake in Type 2 Diabetes Mellitus: Importance of Macro and Micronutrients in Glucose Homeostasis

**DOI:** 10.3390/nu14102132

**Published:** 2022-05-20

**Authors:** Nadia Kheriji, Wided Boukhalfa, Faten Mahjoub, Meriem Hechmi, Thouraya Dakhlaoui, Mehdi Mrad, Afef Hadj Salah Bahlous, Nadia Ben Amor, Henda Jamoussi, Rym Kefi

**Affiliations:** 1 Laboratory of Biomedical Genomics and Oncogenetics, Institut Pasteur de Tunis, University of Tunis El Manar Tunis, Tunis 1002, Tunisia; nadia.kheriji@pasteur.utm.tn (N.K.); boukhalfawided7@gmail.com (W.B.); meriem.hechmi@pasteur.utm.tn (M.H.); 2 Faculty of Medicine of Tunis, University of Tunis El Manar, EL Manar I, Tunis 2092, Tunisia; mehdi.mrad@fmt.utm.tn (M.M.); afef_bahlous@yahoo.fr (A.H.S.B.); 3 National Institute of Nutrition & Food Technology of Tunis, Service “A” of Nutritional Diseases, Tunis 1007, Tunisia; faten_mahjoub@yahoo.fr (F.M.); hendajamoussi@gmail.com (H.J.); 4 Regional Association of Diabetics of Zaghouan-Regional Hospital of Zaghouan, Zaghwān 1100, Tunisia; diab.zaghouan@gmail.com; 5 Laboratory of Clinical Biochemistry and Hormonology, Institut Pasteur de Tunis, Tunis 1002, Tunisia; 6 Research Unit UR18ES01 on “Obesity”, National Institute of Nutrition and Food Technology, Tunis 2092, Tunisia; nadia.benamor@outlook.com

**Keywords:** nutrition, diet, glucose homeostasis, Tunisia, North Africa, vitamins, minerals, protein, 24-h recall

## Abstract

The prevalence of Type 2 diabetes (T2D) is increasing worldwide. Genetics and lifestyle, especially diet, are contributing factors. Analyses of macro- and micronutrient intake across global populations may help to explain their impact on glucose homeostasis and disease development. To this end, 420 Tunisians were enrolled in a prospective cross-sectional study of daily food consumption. Various data were collected and blood samples were drawn for biochemical assay. A 24-h recall questionnaire was obtained from participants to evaluate dietary intake. Statistical analyses were conducted using Nutrilog and R software. Biochemical analyses stratified the studied population (*n =* 371) into three groups: diabetics (*n =* 106), prediabetics (*n =* 192) and controls (*n =* 73); 49 subjects were excluded. Our results showed that Tunisians had hypercaloric diets high in carbohydrates and fat with variability in the levels of some vitamins and minerals, including riboflavin and niacin, that were statistically different among groups. The lower intake of vitamin D was associated with a greater risk of T2D. Higher vitamin A and sodium intake were associated with poor glucose homeostasis, although protein intake may improve it. In perspective, nutrigenomic studies can provide insight into problematic diets and poor eating habits and offer opportunities to analyze the effects of behavioral changes that can mitigate T2D development and progression.

## 1. Introduction

Type 2 Diabetes (T2D) is a serious public health problem worldwide. It is characterized by permanent hyperglycemia associated with impaired insulin secretion or its peripheral sensitivity [1]. According to the International Diabetes Federation (IDF), more than 530 million adults had diabetes in 2021, and over 780 million adults are expected to suffer from diabetes by 2040. Diabetes is a major cause of morbidity and mortality. One person dies every five seconds as a result of this disease [2]. This is a real burden that weighs heavily on public health in terms of care expenditure, especially in developing countries. 

The Middle East and North Africa (MENA) region has the highest diabetes rate (16%) and related death prevalence (24.5%) [2]. In Tunisia, diabetes prevalence has continued to increase dramatically in the last few years, increasing from 15.5% in 2016 [3] to 18% in 2019 [4], and it is estimated to reach 26% by 2027 [5]. 

T2D, the most common form of diabetes, is caused by both genetic and environmental factors [6]. Regrettably, it is unlikely to affect risk factors such as family history, age, or ethnicity, yet improvement in lifestyle risk factors that include physical activity, smoking behavior and eating is possible. Hence, nutrition is a crucial and double-edged sword in the development and management of diabetes. There is ample evidence of the relationship between diet and diabetes development. Functional studies indicated that a hypercaloric diet high in glucose and fatty acids induces epigenetic changes among diabetics [7]. Also, excessive food intake induces stagnation of the mitochondrial machinery leading to the increased reduction of oxygen to superoxide (O_2_^−^), a pathological process incriminated with diabetes etiology. Indeed, excess fatty acids that accumulate in the cytoplasm are known to cause insulin resistance. As a result, this could dysregulate the flow of nutrients to the mitochondria in non-insulin-dependent cells, thus leading to an increased production of free radicals followed by oxidative damage [8]. Moreover, several nutrients have pro-inflammatory action. For example, saturated fatty acids and their derivatives show greater involvement in the activation of inflammasome and the maturation of certain cytokines and interleukins, the main players in the inflammation process [9]. Furthermore, some epidemiological studies have demonstrated the effect of alkali nutrients on the body’s acid balance, which can change insulin sensitivity and glucose homeostasis [10]. As an example, diets rich in protein and poor in fibers may induce the production of metabolic acids and worsen insulin sensitivity [11]. Contrastingly, balanced diets such as the Mediterranean and low-fat diets could help to prevent diabetes complications [12]. In this regard, a randomized clinical trial showed that the administration of a low-carb high-protein diet for six weeks to T2D patients improved the lipid profile and metabolic control of these subjects [13]. Furthermore, a recent study proved the anti-inflammatory and anti-oxidative effects of omega-3polyunsaturated fats in T2D patients. The same study suggested that omega-3 polyunsaturated fat supplementation had a promising role in preventing neuro-inflammatory diseases in these subjects [14].

According to the Global Nutrition Reports of 2021, Tunisia has shown limited progress towards achieving diet-related non-communicable disease (NCD) targets, especially for obesity and diabetes [15]. Based on rapid urbanization and civilization, recent studies have shown a drastic change in the diet of Tunisians in the form of a nutritional transition towards a western diet, and this could enormously increase the prevalence of NCD like _T2D and related complications [16]. Thus, efforts to revive the healthy diet have become a national challenge. Few studies have been conducted in Tunisia to evaluate the relationship between diet composition and the occurrence of metabolic diseases, particularly T2D [17]. However, additional investigations are required for a better understanding of the role of diet in glucose homeostasis. Therefore, the aim of the present study was to determine nutritional components associated with diabetes development and their impact on glucose homeostasis.

## 2. Materials and Methods

### 2.1. Study Design 

This study was performed in the frame of a collaboration between the “Regional Association of Diabetics of Zaghouan” which is a Tunisian Civil Society Organization (CSO) and the Laboratory of Biomedical Genomics and Oncogenetics in Institut Pasteur de Tunis (IPT). In fact, during its awareness-raising campaigns in the six districts of Zaghouan, a North-Eastern region of Tunisia, the regional association of the Diabetics of Zaghouan has noted high prevalence of T2D in the region. Taking this observation into account, a study of the role of dietary intake in diabetes development and glucose homeostasis regulation was proposed.

### 2.2. Type of the Study 

The study is a prospective cross-sectional survey of the nutritional dietary intake in a representative sample of a Northern East Tunisian population. This study was conducted during the two months from June to August of 2020, after the first wave of the COVID_19 emergency. In fact, throughout this study, reliable estimates of food and nutrient intakes were obtained in order to relate them to the health status of individuals. 

### 2.3. Participants

A total of 420 participants were enrolled in this study during diabetes awareness raising campaigns in the region of Zaghouan. The inclusion and exclusion criteria were fixed by researchers as the following: included in the study were all aboriginal inhabitants of the Zaghouan region 30 years old and above. Excluded from the study were all newly discovered diabetics. After obtaining informed consent from all participants, a questionnaire was used to collect demographic (age, gender) and anthropometric data (body mass index, BMI, waist circumference (WC), as well as data related to lifestyle (physical activity, smoking habits and alcohol consumption). A 24-h recall (24HR) nutrition survey was then filled out by the nutritionist from CSO for all the participants. In addition, blood samples were collected to assess the glycated hemoglobin (HbA1c) level. All this data was obtained only once. Based on the HbA1c level and American Diabetes Association (ADA) recommendations, participants were stratified into three groups: diabetic, non-diabetic (controls) and prediabetic. 

### 2.4. Measurements

#### 2.4.1. Dietary Intake 

The dietary intake was assessed using the 24HR questionnaire in order to assess the relationship between diet, diabetes and glucose homeostasis. The 24HR questionnaire yielded detailed information on the number and quantity of meals, foods and beverages consumed for breakfast, lunch and dinner as well as during morning, afternoon and evening snacks. Then, 24HR nutritional surveys were processed with the Nutrilog software (Version 3.20) (Nutrilog Europe Society, Marans, France) available at the Research Unit of Obesity in National Institute of Nutrition & Food Technology of Tunis. The Nutrilog software allows the conversion of food and beverages into energy intake. Added to that, it gives an estimation of the distribution of macro and micronutrients and the daily intake of each nutrient per day. The recommended dietary allowance (RDA) and estimated average requirement (EAR) were determined in accordance with the European nutritional references [18,19]. 

#### 2.4.2. Clinical Data and Biological Analyses

Age, gender, body mass index (BMI), waist circumference (WC), physical activity, smoking habits and alcohol consumption were included in the study as covariates due to their implication with T2D [4]. In accordance with the ADA guidelines [20], diabetes was defined as HbA1c ≥ 6.5%. For prediabetes, the HbA1c level ranges between 5.7 and 6.49%. The control group included participants with HbA1c level less than 5.6%. HbA1C measurements were performed in the laboratory of biochemistry in IPT using certified and standardized methods. Overweight and obesity were defined as BMI ranges of 25–29.9 kg/m^2^ and ≥30 kg/m^2^, respectively [21]. Abdominal obesity was defined as WC ≥ 80 cm for women and WC ≥ 94 cm for men [22]. Waist circumference was measured across the belly button wearing light clothing by trained staff using a measuring tape on the subject while they were standing and breathing normally.

### 2.5. Ethics Approval and Consent to Participate

The survey protocol was reviewed and approved by the Ethical Committee of the Institut Pasteur de Tunis (Registration number IRB00005445, FWA00010074, ref.2019/14/I/LR16IPT/V4). Once participants were informed about the objectives of the study and the data collection process, they signed an informed consent in compliance with the guidelines of the Declaration of Helsinki [23]. 

### 2.6. Statistical Analyses

All collected data (demographic, anthropometric, lifestyle and dietary intake) were integrated into a secure database for statistical analysis using the data mining R software (R Core Team, Auckland, USA) for Windows version 4.0.2 [24]. The results are expressed as mean ± standard deviation for the quantitative variables or as number and frequency distribution for the categorical variables. Moreover, to compare changes in the composition of diet between the three studied groups, a variance analysis was conducted using an ANOVA test. All significant ANOVA tests were followed by Tukey’s post-hoc tests, multiple pairwise comparisons to specify which group was different from the others. A linear logistic regression analysis was then conducted to identify nutrients associated with T2D development and glucose homeostasis among diabetic patients. In order to determine dietary intake, differences between controlled and uncontrolled diabetics, a *t*-test was performed. Statistical significance was set at *p* < 0.05. Bonferroni correction was applied to maintain an overall 0.95 (CI) confidence interval in multiple comparisons for all analyses [25]. Macro and micronutrient intake was the exposure variable for this study. Altogether, 38 nutrients were studied. The outcome variable was T2D.

## 3. Results

### 3.1. Baseline Characteristics 

Baseline characteristics of the subjects included in the study (*n =* 418) are shown in Table 1. Of all participants, 76% (*n =* 317) were women and 24% (*n =* 101) were men. The mean age was about 57.14 ± 13.51 years. Obesity was found in 50% of the participants. Dietary intake was mainly characterized by an excess intake of fat, simple carbohydrates and animal protein while the intake of fibers, vitamins and minerals was insufficient for the studied population. Also, an excessive intake of sodium (more than 4 times the RDA quantity) and iodine was noted (Table 1).

**Figure 1:**
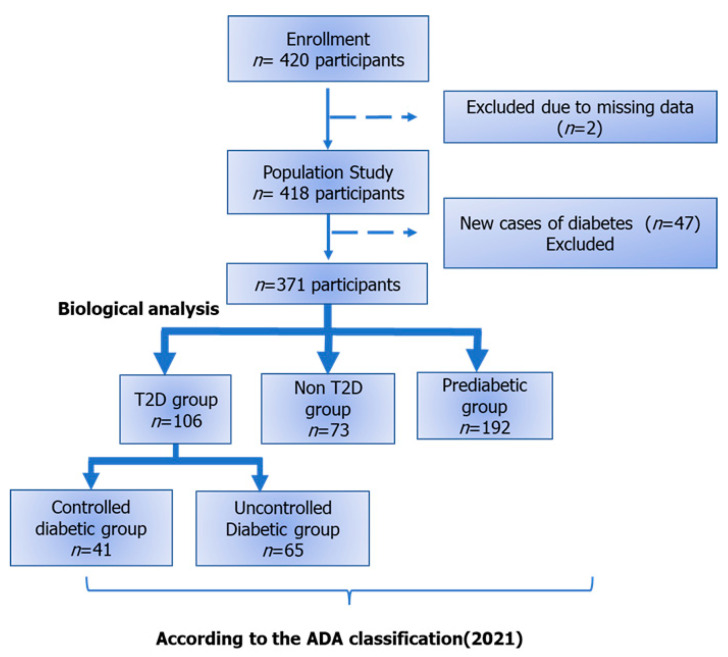
Flowchart of the study.

HbA1c analyses showed that 28% of the participants were T2D (*n =* 106) and 20% (*n =* 73) were not diabetic. Forty-seven newly discovered diabetic patients were identified. Consequently, these individuals were excluded from the comparative analyses since they had not received nutritional education. Hence, the sample included in the study consisted of 371 individuals stratified into three groups: (i) The T2D group: confirmed diabetic participants with HbA1c ≥ 6.5% (*n =* 106); (ii) The non-T2D group or controls: participants who did not have diabetes with an HbA1c level < 5.7% (*n =* 73); and (iii) The prediabetic group: participants who were not previously diagnosed as diabetic with HbA1c ranges between 5.7 and 6.49% (*n =* 192). This classification was based on the HbA1c levels as recommended by the American Diabetes Association (ADA) [20]. Moreover, the T2D group was clustered into two groups; the controlled (HbA1c ≤ 7%) and uncontrolled group (HbA1c > 7%) (Figure 1).

T2D: Type 2 Diabetes; ADA: American Diabetes Association. The controlled diabetic is defined as a diabetic participant with HbA1c level equal to 7% or less during the survey. In the same manner, the uncontrolled diabetic group is defined as diabetic individuals with HbA1c levels above 7% during the study.

### 3.2. Differences in Macro and Micronutrients Intake between Studied Groups 

Comparison of nutritional data was conducted on a total of 371 participants stratified into three groups: diabetics (*n =* 106), prediabetics (*n =* 192), and controls (*n =* 72). The results are described in Table 2. A variance analysis showed significant differences in terms of age, BMI and WC. Similarity was observed in the energy intake of macronutrients, and the only significant differences were observed in the TEI of sugar and fat. Concerning micronutrient intake, differences in vitamin D, riboflavin (vitamin B2), niacin (vitamin B3), vitamin B5, magnesium, calcium, phosphorus, potassium, total iron and zinc intake were found. The three compared groups showed significant differences in cholesterol and water intake as well. 

**Figure 2:**
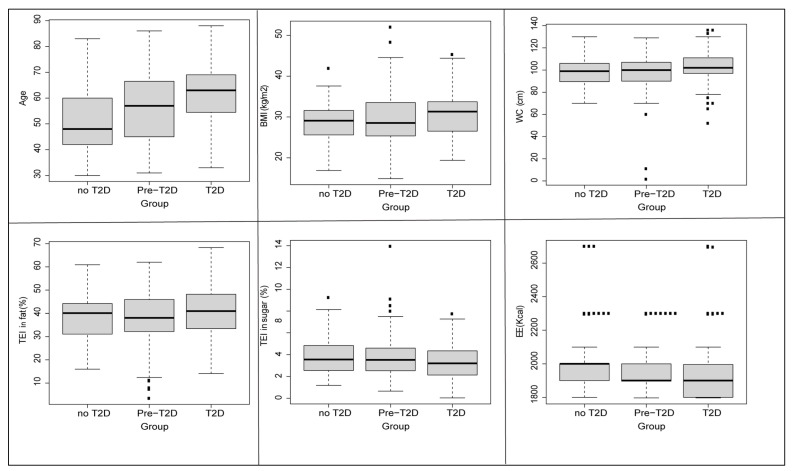
Differences in anthropometric and macronutrients factors between diabetics, controls and prediabetics groups. BMI: Body Mass Index; TEI: Total Energy Intake; WC: Waist Circumference; Pairwise comparisons (*t*-test); The number of the square symbol (▪) in the boxplots reflects the degree of significance of the statistical test.

**Figure 3:**
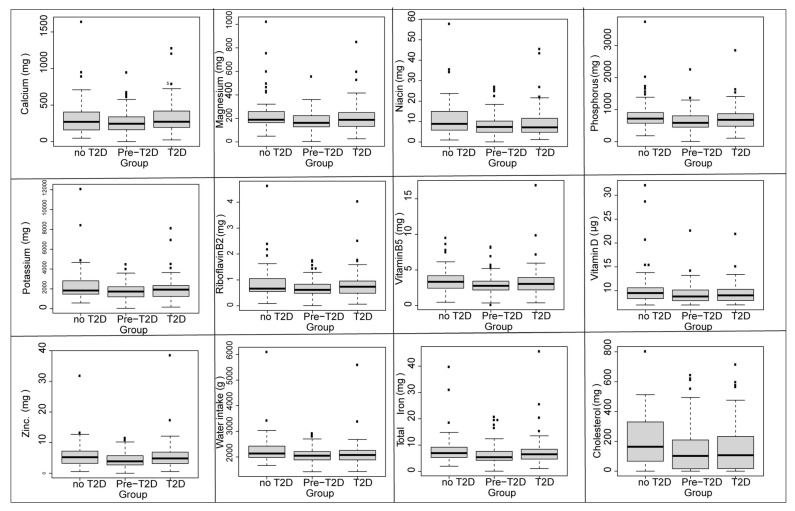
Differences in micronutrients between diabetics, controls and Prediabetic groups. Pairwise comparisons (*t*-test); The number of the square symbol (▪) in the boxplots reflects the degree of significance of the statistical test.

The ANOVA test results were confirmed by Tukey’s post-hoc test as described in Figure 2 and Figure 3.

In the present study, more than 60% of diabetic participants had uncontrolled diabetes. A comparison between controlled and uncontrolled diabetics showed significant differences in terms of TEI of protein (%), riboflavin (vitamin B2) and Niacin (vitamin B3) (Table 3).

A linear regression analysis conducted between T2D and non T2D subjects showed a linear relationship between T2D and dietary intake in vitamin D. However, no association was observed between macronutrients and T2D (Table 4).

With regard to diabetes management and glucose homeostasis, a linear regression analysis comparing the controlled and uncontrolled diabetics showed an association between vitamin A, sodium intake, the TEI of protein and glucose homeostasis. In fact, vitamin A and sodium intake were associated with a poor glucose homeostasis (OR = 0.99, *p*-value = 0.03 and OR = 1, *p*-value = 0.03), respectively, while TEI of protein was associated with a positive glucose regulation (OR = 0.28, *p*-value < 0.05) (Table 5).

## 4. Discussion

The aim of this study was to identify nutritional components associated with T2D development and glucose homeostasis in Tunisian diabetic patients. 

The dietary intake of the Tunisian population was hypercaloric and obesogenic with an excess intake of fat and simple carbohydrates observed in the studied group. In actuality, it is a diet lacking in fiber, vitamins and minerals. An excessive intake of sodium and iodine was also noted. These results are consistent with previous Tunisian studies highlighting changes in dietary behaviors. In fact, the MENA region and particularly Tunisia have experienced a major increase in the prevalence of diabetes, obesity and hypertension, which is due to deviation from the Mediterranean to a westernized diet characterized by fast food consumption [26,27,28]. In this study, the only significant difference observed between men and women was in terms of sugar intake, controversially by Mohamed et al., who showed gender differences in terms of fat, cholesterol, calcium, potassium, phosphorus and vitamin C intake [29].

The comparison between the three studied groups (T2D, controls and pre-T2D) showed significant differences in dietary intake, particularly in sugar and fat. These results confirm previous studies showing that a hypercaloric diet, salt, sugar and fat intake were usually associated with an increased prevalence of chronic diseases such as instance diabetes, obesity, hypertension and cardiovascular diseases [30,31]. Together, a high fat, high carb diet alters glucose tolerance and insulin sensitivity by reducing adipocyte progenitor cells, inducing vascular dysfunction and oxidative stress in adipose tissue arteries [32]. 

The present cross-sectional study also showed a significant difference between the studied groups in terms of micronutrient intake in vitamins including riboflavin, niacin, vitamin B5, and vitamin D as well as some minerals including magnesium, calcium, zinc, phosphorus, potassium, total iron, cholesterol and water intake. It was noted that the highest quantity of vitamins and minerals was found in the control group, suggesting their role in T2D prevention. These findings are in line with other studies showing the deficiency of these micronutrients among diabetics, since an important amount is eliminated in urine daily waste [33]. Therefore, T2D patients need to increase their daily intake of micronutrients [34]. In this context, several studies have demonstrated the strong antioxidant properties of vitamins that might help in improving the immune response and inflammatory processes [35]. A particular example would be riboflavin (Vitamin B2). which has been used in the prevention of a wide array of health diseases like migraine, anemia, cancer, hyperglycemia, hypertension, oxidative stress and diabetes mellitus [36]. The results of the current study support previous findings suggesting that supplementation with dietary riboflavin might help in diabetic complication prevention by decreasing low-grade inflammation caused by oxidative stress [37].

These results are in agreement with Suez et al., who have associated riboflavin with hyperglycemia due to its important role in glucose absorption [38]. However, the supporting research has to be unraveled in this direction to reinforce these findings.

With regard to minerals, there is ample evidence that magnesium, calcium, zinc, phosphorus and potassium are involved in the insulin secretion pathway and, consequently, in the regulation of blood glucose homeostasis [39]. This work showed high levels of magnesium among the control group conferring a protective effect against diabetes. This result is also in accordance with many studies that have confirmed the beneficial role of magnesium in declining oxidative stress and inflammatory actors, two main contributors to insulin resistance [40]. Some studies have suggested that magnesium supplementation may improve the glycemic profile, while others have shown the opposite [41]. These discrepancies in baseline magnesium levels and metabolic control could explain the differences in diverse studies. 

Regression analyses showed that vitamin D was associated with the occurrence of T2D. These findings corroborate previous studies highlighting the strong association between vitamin D and the development of diabetes and its complications [42]. Another study ascertained that vitamin D deficiency alters insulin synthesis and secretion [43]. However, no link between vitamin D and glucose homeostasis has been shown in this study. To the contrary, several studies have emphasized the crucial role of vitamin D in glucose homeostasis on different levels. It enhanced calcium and phosphorus absorption, improved insulin signaling, and it decreases pro-inflammatory pathway activation [44]. In this regard, a report published by the Endocrine Society has concluded that insufficiencies of Vitamin D and calcium negatively influence glucose homeostasis and thus supplementation with both nutrients may be beneficial in diabetes management [45]. 

It is also important to note that vitamin D dietary intake does not meet the RDA threshold (<15 µg/day). This result confirms the observation by Fakhfakh et al. that there is a deficiency of vitamin D among the Tunisian population [46]. 

Concerning glucose homeostasis, our findings underlined the positive effect of protein intake on glucose regulation, while niacin (vitamin B3) and riboflavin intake displayed a negative impact on diabetes management. In addition, several studies have shown benefits of protein intake on clinical indicators of metabolic disease. In fact, a high protein diet improves dyslipidemia and insulin resistance [47]. Yet a recent study has shown that excess amino acids could reduce insulin-mediated glucose via an acid–base imbalance [10,11]. Therefore, with regard to this inconsistency, it is important to pay attention to the type of protein diet and the sources of protein for the prevention of diabetes. In accordance with our results, a recent study conducted in 2017 showed that niacin does not reduce cardiovascular mortality and is associated with side effects [48]. It was also reported that long-term treatment with niacin (vitamin B3) at a low dose induces impaired mitochondrial respiration capacity and energy production in skeletal muscle, with an increased expression of mitophagy and autophagy mediators, which could induce glucose intolerance and lipid accumulation in non-obese mice [49]. Our results contradict other studies that highlight the positive role of niacin in reducing blood sugar, fatty liver and preventing diabetes complications [50,51,52]. 

Taking into account this data, further clinical therapeutic survey trials are needed to determine the appropriate dose and timing of vitamin B3 use as a preventive treatment for T2D complications.

To sum up, the available literature shows conflicting results regarding the role of riboflavin and niacin in T2D development and glucose regulation. Therefore, between those who affirm and deny the positive role of niacin and riboflavin in diabetes management, additional well-designed studies are clearly needed.

Furthermore, it was noted that vitamin A intake was higher in the uncontrolled group compared to the controlled one. The regression analyses also showed an association between vitamin A and poor glucose homeostasis. This result is in accordance with the literature wherein vitamin A intake has been associated with the occurrence of gestational diabetes [53]. In Tunisia, until now, no study has been conducted to determine the role of vitamin A intake in diabetic patients; nevertheless, one study showed that obese T2D women have significantly higher plasma vitamin A than the control group, suggesting the involvement of vitamin A in the impaired regulation of glycemia [54]. Sayyed et al. have also shown the impact of vitamin A intake on the higher risk of metabolic syndrome among diabetic Iranian women [55]. However, other studies have demonstrated that vitamin A is associated with the regulation of glucose homeostasis due to its crucial role in pancreas islet functions and insulin production [56,57]. This disparity in the role of dietary intake in vitamin A could be explained by the heterogeneity of the studied populations in terms of different dietary behaviors, ethnic origins and health status influencing vitamin A metabolism [58]. These controversial results emphasize the importance of conducting well designed experiments with more T2D subjects [59].

In fact, in this study, an excess intake of sodium among the whole study population was found (more than 4 times the RDA). An association between sodium intake and blood glucose homeostasis was observed (OR = 1, *p*-value < 0.03). Our findings are endorsed by Baudrand et al., who have shown the benefit of the sodium intake restriction in insulin resistance improvement throughout increasing adiponectin secretion and reducing pro-inflammatory processes [60]. High dietary salt intake increases cortisol secretion and insulin resistance, i.e poor glucose regulation [61,62]. Despite its role in glycemia dysregulation, sodium supplementation in T2D patients could improve the sympathetic nervous system without altering endothelial function [63]. Thus, this suggests the importance of sodium quantity intake in diabetes management. This implies that personalized salt intake in diabetic patients should be considered for an optimal glucose homeostasis [64].

These results could be of use for health care providers in dietary management to reduce the incidence of this metabolic disease and its consequences. Indeed, we highly suggest the studied population (i) to increase their daily intake of proteins and fibers, (ii) to reduce their intake of foods high in fat and salt, (iii) to acquire a diet rich in vitamin B2 and B3 and finally (iv) to improve vitamin D status in the population at risk for diabetes by enriching their diet composition with this vitamin. 

The 24-h recall method that tracks food intake for the past 24 h is a simple method to use and is recommended for carrying out food surveys on a large population scale. However, this method does not represent variations in food intake over the week. Indeed, it is ideally recommended that one combine food survey methods (eg: the food history method) in order to obtain a detailed food intake throughout the week. However, the combination of these methods is not easy on a large scale because it is difficult to implement (takes a lot of time) and can lead to a selection bias.

To the best of our knowledge, this study is the first of its kind investigating the role of micro and macronutrient intake on glucose homeostasis among the Tunisian population. It provides data regarding the role of vitamin A, sodium and protein intake on glucose homeostasis. Since the conflicting data reported in the literature, the present study will enrich debates concerning the role of these nutrients on glucose regulation. In addition, the present study includes prediabetic subjects and provides new insights towards T2D prevention. In fact, our results will help to establish a National of Diabetes Prevention Program (DPP) adapted to the nutritional specificities of the Tunisian population. The findings will give valuable information to groups involved in making policies for the health sector in intensifying lifestyle and dietary measures. Finally, this study highlights the importance of collaboration between CSO and researchers to bridge the gap between public health innovation and scientific knowledge sharing. 

## 5. Conclusions

The regulation of glucose homeostasis in diabetics is strongly linked to the quantity and quality of dietary intake. Our data confirm that vitamin D insufficiency increased the risk of T2D among Tunisians. This study is in line with others suggesting the negative effect of vitamin A and sodium intake on glucose homeostasis. On the other hand, it seems that protein intake might improve glycaemia levels. This study provides data which could help health-care professionals provide clear information about the linkage of diet with T2D and hence establish practical guidelines for dietary regimens to prevent or delay the onset of diabetes. 

The importance of this study lies in the inclusion of the prediabetic group, which provides new insights towards T2D prevention. Furthermore, the heterogeneous genetic landscape of the Tunisian population makes it a unique model for carrying out nutrigenomic and nutrigenetic studies. Finally, the present study will enhance knowledge with regard to the effect of dietary intake on glucose homeostasis.

## Figures and Tables

**Table 1:** Baseline characteristics of the study population by gender (*n =* 418).

	All *n =* 418 (100%) Mean ± SD	Women *n =* 317 (76%) Mean ± SD	MEN *n =* 101 (24%) Mean ± SD	*p*-Value	Reference Range (RDA)
	Anthropometric data				
Age	57.14 ± 13.51	55.97 ± 13.05	60.89 ± 14.34	**0.002**	-
BMI (kg/m^2^)	30.03 ± 5.95	30.98 ± 5.93	27.02 ± 4.95	**<10^−3^**	18.5–24.9
WC (cm)	100.80 ± 14.66	101.43 ± 14.72	98.77 ± 4.38	0.109	F: 80 M: 94
	Life style data				
Physical activity, n (%)	Yes: 191 (45%) No: 227 (55%)	Yes:139 (33.25%) No: 178 (42.58%)	Yes: 52 (12.44%) No: 49 (11.72%)	0.179	-
Smoking habits, n (%)	Yes: 65 (16%) No: 353 (84%)	Yes: 11 (2.63%) No: 306 (73.17	Yes: 54 (13%) No: 47 (11.2%)	**<10^−3^**	-
Alcoholism status, n (%)	Yes: 2 (0.48%) No:416 (99.52%)	Yes: 2 (0.47%) No: 315 (75.35%)	Yes: 1 (0.24%) No: 100 (23.92%)	0.709	-
	Energy Intake from macronutrients				
TEI (Kcal)	1766.3 ± 729.45	1738.58 ± 744.19	1853.32 ± 677.30	0.149	F: 2000 M: 2500
TEI of Protein (g/Kg/day)	0.97 ± 0.49	0.99 ± 0.51	0.92 ± 0.43	0.143	0.83–1
TEI of Protein (%)	12.450 ± 4.79	12.38 ± 4.91	12.66 ± 4.41	0.589	10–20
TEI of Animal Protein (%)	30.89 ± 7.616	33.16 ± 7.83	22.67 ± 6.95	0.793	25
AP/PP	0.7617 ± 0.99	0.76 ± 1.00	0.78 ± 0.98	0.873	0.33
TEI of Fat (g/Kg/day)	1.40 ± 0.69	1.42 ± 0.72	1.31 ± 0.60	0.139	0.7–1
TEI of Fat (%)	38.75 ± 10.82	38.61 ± 10.83	39.19 ± 10.86	0.640	33–35
TEI of Saturated Fat (%)	6.162 ± 2.65	6.29 ± 2.63	5.78 ± 2.69	0.148	12
TEI of Monounsaturated Fat (%)	17.00 ± 8.03	17.24 ± 8.14	16.25 ± 7.69	0.334	15–20
TEI of Polyunsaturated Fat (%)	7.66 ± 5.34	7.62 ± 5.75	7.778 ± 3.83	0.782	5
TEI of Sugar (g/Kg/day)	3.67 ± 0.72	3.79 ± 1.82	3.29 ± 1.30	**0.002**	4–5
TEI of Simple Carbohydrates (%)	11.29 ± 8.73	11.45 ± 7.90	3.29 ± 10.95	0.647	<10
TEI of Sugar (%)	46.00 ± 10.00	46.00 ± 10.00	45.00 ± 10.00	0.467	50 to 55
Dietary fiber (g)	19.00 ± 13.00	18.00 ± 14.00	19.00 ± 16.00	0.823	25–30
Cholesterol (mg)	152 ± 150	150 ± 144	157 ± 168	0.711	300
	Energy Intake from Micronutrients (Vitamins and Minerals)				
Vitamin A (µg)	89.00 ± 167.00	90.00 ± 73.00	87.00 ± 151.00	0.865	-
Vitamin D (µg)	9.00 ± 2.00	9.00 ± 2.94	9.54 ± 2.08	0.963	15
Vitamin E (mg)	18.00 ± 11.00	18.00 ± 10.00	20.00 ± 13.00	0.138	F: 9 M: 10
Vitamin C (mg)	44.00 ± 37.00	42.00 ± 32.00	51.00 ± 50.00	0.152	-
Vitamin B1 (mg)	2.00 ± 22.00	2.00 ± 25.00	0.61 ± 0.28	0.315	-
Vitamin B2 (Riboflavin) (mg)	0.74 ± 0.46	0.75 ± 0.50	0.71 ± 0.33	0.476	-
Vitamin B3 (Niacin) (mg)	9.1 ± 6.87	9.20 ± 6.9	8.75 ± 6.58	0.604	10
Vitamin B5 (mg)	3.00 ± 1.63	3.00 ± 1.69	3.00 ± 1.42	0.917	F: 5 M: 6
Vitamin B6 (mg)	0.98 ± 0.62	0.98 ± 0.64	0.97 ± 0.56	0.821	-
Vitamin B9 (Folate) (µg)	167 ± 167	166 ± 182	171 ± 110	0.758	-
Vitamin B12(µg)	2.00 ± 0.900	2.00 ± 1.00	2.00 ± 1.00	0.171	4
Magnesium (mg)	196 ± 10.00	194 ± 116	201 ± 90	0.615	F:300–360 M:380–420
Calcium (mg)	296 ± 194	296 ± 200	293 ± 176	0.897	-
Phosphorus (mg)	701 ± 381	700 ± 398	705 ± 328	0.899	550–700
Potassium (mg)	1947 ± 1149	1927 ± 1182	2008 ± 1047	0.568	3500
Sodium (mg)	7568 ± 3871	7492 ± 4125	7805 ± 2964	0.465	1500
Total Iron (mg)	6.94 ± 4.67	6.92 ± 4.98	6.98 ± 3.56	0.908	-
Zinc (mg)	5.00 ± 3.00	4.00 ± 3.00	5.00 ± 2.00	0.327	-
Copper (mg)	4.00 ± 53.00	3.00 ± 35.00	10 ± 89	0.452	F: 1.5 M: 1.9
Manganese (mg)	2.00 ± 1.00	2.00 ± 1.00	2.00 ± 1.00	0.189	F: 2.5 M: 2.8
Iodide (µg)	209 ± 181	207.00 ± 191.00	215 ± 148	0.697	150
Selenium (µg)	85.00 ± 62.00	82.00 ± 57.00	97.00 ± 73.00	0.104	-
Water intake (g)	2123 ± 425	2100.8 ± 434	2192.01 ± 390	0.083	2500

F: Females; M: Males; BMI: Body Mass Index, WC: Waist Circumference, TEI: Total Energy Intake, AP/PP: Animal Protein/Plant Protein, significant *p*-value < 0.05 are in bold.

**Table 2:** Differences in energy intake in macro and micronutrients among study population (*n =* 371).

	Diabetic Group *n* = 106 (28%)	Control Group *n* = 73 (20%)	Prediabetic Group *n* = 192 (52%)	F-Value	Adjusted *p*-Value
	F = 72(69%) M = 33(31%)	F = 53 (73%) M = 20 (27%)	F = 158(82%) M = 34 (17%)		
	Anthropometric data				
Age	61.42	51.36	56.90	13.13	**<10^−3^**
BMI (kg/m^2^)	30.84	28.61	29.75	3.28	**0.03**
WC (cm)	F = 104.76 M = 100.42	F = 98.44 M = 96.25	F = 98.61 M = 95.14	5.62	**<10^−3^**
	Energy Intake from macronutrients				
TEI (Kcal)	1734.08	1896.26	1737.08	1.44	0.23
TEI of Protein (g/Kg/day)	0.96	1.05	0.94	1.21	0.29
TEI of Protein (%)	12.52	13.04	12.21	0.78	0.45
TEI of Animal Protein (%)	4.43	12.69	8.39	1.12	0.32
AP/PP	0.71	1.07	0.71	1.64	0.19
TEI of Fat (g/Kg/day)	1.39	1.40	1.39	10^−3^	0.99
TEI of Fat (%)	41.03	38.09	37.86	3.09	**<0.05**
TEI of Saturated Fat (%)	6.19	5.98	6.18	0.16	0.84
TEI of Monounsaturated Fat (%)	18.29	16.15	16.93	1.35	0.26
TEI of Polyunsaturated Fat (%)	7.83	8.26	7.51	0.39	0.67
TEI of Sugar (g/Kg/day)	3.39	3.86	3.77	2.39	0.09
TEI of Simple Carbohydrates (%)	10.28	11.67	12.02	1.01	0.36
TEI of Sugar (%)	43.75	46.24	47.31	4.09	**<0.05**
Dietary fiber (g)	20.20	20.60	17.96	1.02	0.36
Cholesterol (mg)	155.63	204.22	134.53	3.81	**0.02**
	Energy Intake from Micronutrients (Vitamins and Minerals)				
Vitamin A (µg)	101.67	85.14	90.57	0.17	0.84
Vitamin D (µg)	9.46	10.58	9.22	5.34	**<10^−3^**
Vitamin E (mg)	20.62	19.05	17.66	1.75	0.17
Vitamin C (mg)	46.69	53.33	40.67	2.51	0.08
Vitamin B1 (mg)	5.31	0.72	0.57	1.20	0.30
Vitamin B2 (Riboflavin) (mg)	0.82	0.88	0.65	5.74	**<10^−3^**
Vitamin B3 (Niacin) (mg)	9.32	11.72	8.24	3.73	**0.02**
Vitamin B5 (mg)	3.31	3.61	2.93	4.23	**0.01**
Vitamin B6 (mg)	0.99	1.17	0.91	2.43	0.09
Vitamin B9 (Folate) (µg)	178.43	214.28	147.44	2.87	0.06
Vitamin B12 (µg)	3.95	2.58	1.50	1.47	0.23
Magnesium (mg)	206.44	235.37	179.36	4.30	**0.01**
Calcium (mg)	228.06	327.64	270.92	2.99	**0.05**
Phosphorus (mg)	719.51	841.70	650.75	3.60	**0.03**
Potassium (mg)	2059.51	2344.87	1777.97	4.17	**0.01**
Sodium (mg)	7928.18	7779.67	7338.97	0.70	0.49
Total Iron (mg)	7.36	8.25	6.29	3.37	**0.03**
Zinc (mg)	5.62	6.03	4.41	5.27	**<10^−3^**
Copper (mg)	10.03	0.84	4.58	0.95	0.38
Manganese (mg)	2.34	2.42	2.06	1.60	0.20
Iodide (µg)	223.75	229.39	198.24	1.15	0.31
Selenium (µg)	86.91	99.30	80.96	1.76	0.17
Water intake (g)	2127.15	2282.41	2070.31	3.32	**0.03**

RDA: Recommended Dietary Allowance, F: Females; M: Males; BMI: Body Mass Index, WC: Waist Circumference, TEI: Total Energy Intake, AP/PP: Animal Protein/Plant Protein, significant *p*-value < 0.05 are in bold. The F-statistic is simply a ratio of two standard deviations that is determined by an ANOVA test. It determines the significance of the groups of variables.

**Table 3:** Differences in total energy intake (TEI) between controlled and uncontrolled diabetics (*n =* 106).

	For Controlled Diabetics (HbA1c ≤ 7%) *n =* 41 (38.67%) Mean ± SEM	For Uncontrolled Diabetics (HbA1c > 7%) *n =* 65 (61.32%) Mean ± SEM	Adjusted *p*-Value
	F = 30 (75%) M = 11 (25%)	F = 42 (65%) M = 23 (35%)	
Anthropometric data			
Age	62.60 ± 1.96	60.65 ± 1.27	0.40
BMI (kg/m^2^)	30.21 ± 0.72	31.24 ± 0.71	0.31
WC (cm)	100.34 ± 2.49	105.27 ± 1.43	0.08
Energy Intake from macronutrients			
TEI (Kcal)	1867.67 ± 11.56	1649.81 ± 80.92	0.11
TEI of Protein (g/Kg/day)	0.97 ± 0.07	0.96 ± 0.08	0.42
TEI of Protein (%)	12.57 ± 0.58	12.51 ± 0.68	**<0.05**
TEI of Animal Protein (%)	3.63 ± 0.54	4.96 ± 0.61	0.15
AP/PP	0.61 ± 0.11	0.78 ± 0.14	0.42
TEI of Fat (g/Kg/day)	1.56 ± 0.15	1.29 ± 0.08	0.11
TEI of Fat (%)	41.37 ± 1.59	40.81 ± 1.31	0.78
TEI of Saturated Fat (%)	6.06 ± 0.43	6.27 ± 0.39	0.60
TEI of Monounsaturated Fat (%)	18.84 ± 1.54	17.93 ± 1.33	0.60
TEI of Polyunsaturated Fat (%)	8.78 ± 0.76	7.20 ± 0.55	0.06
TEI of Sugar (g/Kg/day)	3.64 ± 0.23	3.23 ± 0.20	0.19
TEI of Simple Carbohydrates (%)	10.26 ± 1.29	10.30 ± 0.87	0.97
TEI of Sugar (%)	44.92 ± 1.37	43.01 ± 1.23	0.30
Dietary fiber (g)	19.48 ± 1.81	20.68 ± 2.27	0.69
Cholesterol (mg)	124.88 ± 21.34	176.12 ± 21.36	0.14
Energy Intake from micronutrients (Vitamins and Minerals)			
Vitamin A (µg)	101.67 ± 9.65	102.57 ± 34.03	0.14
Vitamin D (µg)	9.92 ± 0.72	9.15 ± 0.50	0.16
Vitamin E (mg)	23.54 ± 2.37	18.67 ± 1.74	0.10
Vitamin C (mg)	41.76 ± 4.64	49.98 ± 7.17	0.40
Vitamin B1 (mg)	5.31 ± 0.05	5.37 ± 6.12	0.31
Vitamin B2 (Riboflavin) (mg)	0.82 ± 0.06	0.83 ± 0.08	**0.02**
Vitamin B3 (Niacin) (mg)	9.32 ± 0.84	9.33 ± 1.12	**0.03**
Vitamin B5 (mg)	2.98 ± 0.24	3.53 ± 0.33	0.18
Vitamin B6 (mg)	0.85 ± 0.08	1.08 ± 0.10	0.08
Vitamin B9 (Folate) (µg)	164.28 ± 16.89	187.87 ± 19.47	0.38
Vitamin B12 (µg)	6.66 ± 4.23	2.14 ± 0.24	0.38
Magnesium (mg)	204.22 ± 18.43	207.93 ± 17.97	0.88
Calcium (mg)	299.26 ± 25.92	347.27 ± 33.16	0.26
Phosphorus (mg)	653.88 ± 59.13	763.27 ± 61.09	0.17
Potassium (mg)	2053.38 ± 166.19	2063.60 ± 186.51	0.96
Sodium (mg)	7928.00 ± 1183.43	7928.18 ± 529.97	0.13
Total Iron (mg)	6.63 ± 0.58	7.85 ± 0.85	0.26
Zinc (mg)	4.89 ± 0.46	6.11 ± 0.67	0.16
Copper (mg)	0.77 ± 0.06	16.20 ± 12.11	0.32
Manganese (mg)	2.36 ± 0.22	2.33 ± 0.26	0.91
Iodide (µg)	280.46 ± 53.41	185.95 ± 17.50	0.14
Selenium (µg)	101.54 ± 11.63	77.17 ± 7.35	0.09
Water intake (g)	2126.17 ± 132.80	2127.80 ± 126.57	0.98

SEM: Standard Error of the Mean; F: Females; M: Males; BMI: Body Mass Index, WC: Waist Circumference, TEI: Total Energy Intake, AP: Animal Protein, PP: Plant Protein, significant *p*-value < 0.05 are in bold.

**Table 4:** Results of linear regression between macro and micronutrients intake and T2D disease.

Covariates	OR (CI) 95%	*p*-Value
Energy Intake from Macronutrients		
TEI (Kcal)	1.00 (1.00–1.00)	0.14
TEI of Protein (g/Kg/day)	0.88 (0.43–1.78)	0.7
TEI of protein (%)	0.77 (0.52–1.10)	0.2
TEI of Animal Protein (%)	0.97 (0.90–1.00)	0.5
AP/PP	0.79 (0.58–1.04)	0.11
TEI of Fat (g/Kg/day)	1.34 (0.80–2.32)	0.3
TEI of Fat (%)	0.80 (0.55–1.13)	0.2
TEI of Saturated Fat (%)	1.01 (0.86–1.19)	>0.9
TEI of Monounsaturated Fat (%)	1.03 (0.98–1.08)	0.3
TEI of Polyunsaturated Fat (%)	0.99 (0.93–1.04)	0.6
TEI of Sugar (g/Kg/day)	0.81 (0.63–1.02)	0.07
TEI of Sugar (%)	0.77 (0.52–1.10)	0.2
TEI of Simple Carbohydrates (%)	0.99 (0.94–1.03)	0.6
Dietary fiber (g)	1.00 (0.98–1.02)	>0.9
Cholesterol (mg)	1.00 (1.00–1.00)	**0.05**
Energy Intake from Micronutrients (Vitamins and Minerals)		
Vitamin A (µg)	1.00 (1.00–1.00)	0.5
Vitamin D (µg)	0.87 (0.73–0.98)	**0.05**
Vitamin E (mg)	1.02 (0.99–1.05)	0.3
Vitamin C (mg)	1.00 (0.99–1.01)	0.6
Vitamin B1 (mg)	1.01 (0.99-NA)	0.8
Vitamin B2 (Riboflavin) (mg)	0.92 (0.44–1.74)	0.8
Vitamin B3 (Niacin) (mg)	0.95 (0.86–1.04)	0.3
Vitamin B5 (mg)	1.09 (0.81–1.49)	0.6
Vitamin B6 (mg)	1.04 (0.32–3.25)	0.9
Vitamin B9 (Folate) (µg)	1.00 (1.00–1.00)	0.9
Vitamin B12 (µg)	1.01 (0.98–1.09)	0.6
Magnesium (mg)	1.00 (0.99–1.00)	0.4
Calcium (mg)	1.00 (1.00–1.00)	0.09
Phosphorus (mg)	1.00 (1.00–1.00)	0.2
Potassium (mg)	1.00 (1.00–1.00)	0.4
Sodium (mg)	1.00 (1.00–1.00)	0.7
Total Iron (mg)	0.95 (0.83–1.07)	0.4
Zinc (mg)	1.04 (0.89–1.22)	0.7
Copper (mg)	1.00 (1.00-NA)	0.8
Manganese (mg)	0.97 (0.76–1.20)	0.8
Iodide (µg)	1.00 (1.00–1.00)	>0.9
Selenium (µg)	1.00 (0.99–1.00)	0.2
Water intake (g)	1.00 (1.00–1.00)	0.2

OR: Odds Ratio, CI: Confidence Interval, significant *p*-value < 0.05 are in bold, AP/PP: Animal Protein/Plant Protein.

**Table 5:** Linear relationship between macro and micronutrients intake and glucose homeostasis.

Covariates	OR (IC) 95%	*p*-Value
Energy Intake from Macronutrients		
TEI (Kcal)	1.00 (1.00–1.00)	0.11
TEI of Protein (g/Kg/day)	0.28 (0.08–0.78)	**0.02**
TEI of Protein (%)	0.96 (0.92–1.01)	0.9
TEI of Animal Protein (%)	0.99 (0.97–1.02)	0.6
AP/PP	0.83 (0.46–1.30)	0.5
TEI of Fat (g/Kg/day)	1.84 (0.95–3.75)	0.07
TEI of Fat (%)	1.08 (0.65–1.85)	0.8
TEI of Saturated Fat (%)	0.96(0.89–1.04)	0.4
TEI of Monounsaturated Fat (%)	1.00 (0.96–1.03)	>0.9
TEI of Polyunsaturated Fat (%)	1.12 (1.00–1.27)	0.06
TEI of Sugar (g/Kg/day)	1.32 (0.92–1.93)	0.14
**TEI of Sugar (%)**	1.10 (0.65–1.93)	0.7
TEI of Simple Carbohydrates (%)	1.00 (0.93–1.06)	>0.9
Dietary fiber (g)	1.04 (0.98–1.10)	0.8
Cholesterol (mg)	1.00 (0.99–1.00)	0.09
Energy Intake from Micronutrients (Vitamins and Minerals)		
Vitamin A (µg)	0.99 (0.98–1.00)	**0.03**
Vitamin D (µg)	1.27 (0.99–1.77)	0.10
Vitamin E (mg)	1.03 (0.99–1.08)	0.12
Vitamin C (mg)	1.00 (0.99–1.02)	>0.9
Vitamin B1 (mg)	0.99 (0.99–1.04)	>0.7
Vitamin B2 (Riboflavin) (mg)	0.28 (0.07–0.93)	0.06
Vitamin B3 (Niacin) (mg)	0.90 (0.77–1.04)	0.2
Vitamin B5 (mg)	1.10 (0.70–1.72)	0.7
Vitamin B6 (mg)	1.12 (0.18–6.87)	0.9
Vitamin B9 (Folate) (µg)	1.00 (0.99–1.00)	0.2
Vitamin B12 (µg)	1.02 (0.99-NA)	0.4
Magnesium (mg)	1.00 (1.00–1.01)	0.3
Calcium (mg)	1.00 (1.00–1.00)	0.7
Phosphorus (mg)	1.00 (1.00–1.00)	0.09
Potassium (mg)	1.00 (1.00–1.00)	0.09
Sodium (mg)	1.00 (1.00–1.00)	**0.03**
Total Iron (mg)	0.98 (0.79–1.17)	0.8
Zinc (mg)	0.87 (0.67–1.12)	0.3
Copper (mg)	0.73 (0.08–0.90)	0.8
Manganese (mg)	1.01 (1.00–1.72)	>0.9
Iodide (µg)	1.00 (1.00–1.01)	0.2
Selenium (µg)	1.01 (1.00–1.01)	0.12
Water intake (g)	1.00 (1.00–1.00)	>0.9

OR: Odds Ratio, CI: Confidence Interval, significant *p*-value < 0.05 are in bold, AP/PP: Animal Protein/Plant Protein.

## Data Availability

Not applicable.
